# Neuropsychological Determinants of Impairment in Activities of Daily Living in Patients With Lewy Body Dementia

**DOI:** 10.7759/cureus.70762

**Published:** 2024-10-03

**Authors:** Nidhi Desai, Huma Nawaz, Nitai D Mukhopadhyay, Kathryn Wyman-Chick, Sarah K Lageman, Ahmed Negida, Matthew J Barrett

**Affiliations:** 1 Department of Neurology, Virginia Commonwealth University, Richmond, USA; 2 School of Population Health - Biostatistics, Virginia Commonwealth University School of Medicine, Richmond, USA; 3 Department of Neurology, HealthPartners, St. Paul, USA

**Keywords:** activities of daily living, dementia with lewy bodies, lewy body dementia, parkinson's disease dementia, processing speed

## Abstract

Background

Understanding the specific cognitive domains associated with activities of daily living (ADLs) impairment in Lewy body dementia (LBD) may help identify which targeted therapeutic interventions to pursue in future research. This study aimed to investigate the neuropsychological determinants of impairment in ADLs in LBD patients.

Methods

We conducted a cross-sectional study of LBD patients referred for a clinical neuropsychological evaluation within the Parkinson’s Disease and Movement Disorders Center at Virginia Commonwealth University. The presence or absence of impairment in eight ADLs and neuropsychological test performances were collected and analyzed. Cluster analysis and hierarchical analysis were used to define ADL impairment into mild, moderate, and severe ADL impairment groups. We then compared cognitive performance between the ADL groups.

Results

This study included 193 LBD patients. Compared to LBD patients with mild and moderate ADL impairment, those with severe ADL impairment had worse scores in global cognition as measured by the Dementia Rating Scale-2 (DRS-2) (p=0.002), speeded visuospatial processing as measured by the Trail Making Test A (p=0.001), speeded executive functioning as measured by the Trail Making Test B (p=0.006), and psychomotor processing speed (p<0.001). Impairments in driving and self-care were associated with worse performances on Trail Making Test A, Trail Making Test B, and psychomotor processing speed (all p<0.05).

Conclusions

In patients with LBD, impaired speeded tasks of visual processing and executive functioning are associated with impaired ADLs, particularly driving and self-care. In order to improve ADLs in LBD, future studies should focus on identifying therapies that improve processing speed performance.

## Introduction

Dementia with Lewy bodies (DLB) and Parkinson’s disease dementia (PDD) together comprise Lewy body dementia (LBD), the third most common cause of dementia [1]. While these conditions can be differentiated based on the relative onset of parkinsonism and dementia, they share the same neuropathology, clinical manifestations, and treatment [2,3]. As both DLB and PDD are dementia syndromes, they require impairment in activities of daily living (ADLs) as a result of cognitive impairment [4,5]. As might be expected, greater cognitive impairment in DLB and PDD is associated with greater impairment in the ability to perform instrumental ADLs [6,7].

Compared to AD, individuals with DLB have been shown to have a more rapid decline in cognition and concomitant function as measured by ADLs [8], and they are more dependent on their caregivers for basic ADLs [9]. This greater impairment in the ability to perform ADLs for DLB patients is associated with a worse quality of life for patients and caregivers and a greater caregiver burden [7,10,11]. Thus, it is important to identify which factors, i.e., cognitive deficits, are associated with impairment in ADLs in DLB and PDD. Identifying which cognitive deficits result in impairment in ADLs may identify which areas of cognition are most important to target with therapeutic interventions. 

Across various neurological conditions, including Alzheimer’s disease, it was previously found that impairment in executive function was associated with impairment in ADLs [12,13]. Impairment in executive function has been reported to be associated with impairment in ADLs in PDD [14], but many other cognitive domains have been implicated, namely attention [6,15,16], particularly visual [17], processing speed [14,18], working memory [18], visual-construction [16], memory [6,14], and language [6]. Considering these disparate findings, there is a need to further investigate which cognitive domains when impaired are associated with greater impairment in ADLs in LBD. Moreover, many studies have sought to address this question in PDD, but a similar literature is not available for DLB.

Further identification of which cognitive deficits are associated with impaired ADLs is needed to improve ADLs and quality of life in those with LBD. To try to clarify the current findings to date for PDD and address the gap in the current literature for DLB, the objective of this study was to investigate the neuropsychological determinants of ADL impairment in LBD patients. Identifying which cognitive domain or domains are associated with impairment in ADLs in LBD can guide the development of future interventional trials in this population to target the cognitive domains most likely to result in functional improvement. Based on the available literature on PDD and other dementias we hypothesized that impairment in executive function and attention, both domains commonly affected in LBD, would be associated with impairment in ADLs.

## Materials and methods

We conducted an observational cross-sectional study for patients seen at Virginia Commonwealth University Parkinson’s Disease and Movement Disorders Center between June 2011 to October 2021. This study was conducted in accordance with the principles of the Declaration of Helsinki and is reported according to the Strengthening the Reporting of Observational Studies in Epidemiology (STROBE) checklist guidelines [19]. The study was approved by the Virginia Commonwealth University Institutional Review Board (IRB approval number HM20021563). All patients gave informed consent before participating in the study.

Participants

We included all patients who completed a baseline neuropsychological evaluation for clinical purposes and consented to have their data used for research. Eligibility for the study included a diagnosis of PDD or DLB as determined by a movement disorders specialist and was confirmed by a neuropsychologist. PDD and DLB were differentiated based on the onset of dementia in relation to parkinsonism if it was present. Patients with DLB were those with parkinsonism that began within one year of dementia and patients with PDD had parkinsonism for greater than one year before developing dementia. We calculated the duration of dementia by determining the time between the date of dementia diagnosis and the date of neuropsychological evaluation. Patients were also required to have comprehensive neuropsychological testing, enrollment in the clinic’s research registry, and be able to read and respond in English.

Assessment of activities of daily living and cognition

Information regarding ADLs was retrieved from the clinical interview portion of participants’ neuropsychological evaluations. The clinical interview obtained the perspectives of the patient and family members at a single time point on the day of the evaluation. All patients and family members were jointly asked if the patient was independent or required assistance with eight ADLs (i.e., Do you manage self-care independently or require assistance?). Research assistants then coded the clinical documentation of responses for each domain dichotomously, as either indicating impairment or not, for the purpose of these analyses. The eight ADLs evaluated consisted of the following: self-care, cooking, household chores, appointments, transportation, finances, medications, and driving. Assessment of self-care included all basic ADLs.

Cognitive performance was obtained from participants’ comprehensive neuropsychological evaluations. Age-based norms and education-based norms were used if available [20,21]. All tests administered to 60 or more patients were included in the analyses. Cognitive assessments were organized by cognitive domain into cognitive screening tests and tests assessing psychomotor processing speed, visuospatial skills, naming, attention, learning, memory, and executive function domains. In participants with one or more neuropsychological tests in the psychomotor processing speed domain, a processing speed composite z-score was created by combining the three processing speed measures assessed in this cohort (i.e., the Wechsler Adult Intelligence Scale-IV (WAIS-IV) symbol search scaled score, WAIS-IV coding scaled score, and Repeatable Battery for the Assessment of Neuropsychological Status (RBANS) coding z-score). Participants were administered one of three depression questionnaires, the Patient Health Questionnaire-9 (PHQ-9), the Beck Depression Inventory-II (BDI-II), or the Geriatric Depression Scale Short Form (GDS-S). To allow these results to be compared between participants, standard cutoffs for the presence of mild depression or greater were applied for each scale (PHQ-9 ≥ 5 [22], BDI-II ≥ 14 [23], and GDS-S ≥ 5 [24]).

Statistical analysis

Statistical analysis was performed using Stata IC 14 (StataCorp. 2015. Stata: Release 14. Statistical Software. College Station, TX: StataCorp LLC.). Both K-means cluster analysis and hierarchical analysis were performed to determine the optimal grouping of patients based on which ADLs were impaired. Following these analyses, we compared neuropsychological assessment scores among mild, moderate, and severe ADL impairment groups using one-way ANOVA for assessment scores that showed normal distribution and Kruskal Wallis tests for scores with non-normal distribution. P-values were corrected for the false discovery rate using the Benjamini-Hochberg procedure to compare cognitive test scores between the three ADL groups. Post hoc t-tests or Wilcoxon rank-sum tests were performed for tests with p<0.0067 on ANOVA or the Kruskal-Wallis test. As secondary analyses, for only those tests found to be different between the ADL impairment groups, we compared Trail Making Test A T-score, Trail Making Test B T-score, and the composite psychomotor processing speed z-score between those with and without impairment in each of the eight ADLs using two-sample t-tests. We performed these secondary analyses to determine which ADL domains were associated with differences in cognitive performance. Statistical significance for these analyses was set at p<0.05.

## Results

Characteristics of the study population

Table 1 shows the clinical characteristics of our study cohort, which included 193 LBD patients, 47 with DLB and 146 with PDD. The mean (SD) age of the total cohort was 73.6±7.9 years. There was no difference between the mean age of DLB and PDD participants (73.4±8.1 and 73.7±7.8 years, p=0.84). Women comprised 42.5% of the total cohort. There was no difference between the percentage of women in the DLB and PDD groups (42.6% vs. 42.3%, p=0.99). The cohort was 81.3% White, 16.6% Black or African American, and 2.1% Asian; 99.3% were not Hispanic or Latino. The median years of education was 14 (interquartile range 12-16). There were 47.9% (89/186) with at least mild levels of depression. Of the 193 LBD patients, 11 had repeat neuropsychological evaluations, which resulted in 207 total time points with ADL assessments and neuropsychological assessments.

**Table 1:** Characteristics of the study population. DLB: dementia with Lewy bodies, PDD: Parkinson’s disease dementia, IQR: interquartile range.

	Total (n=193)	DLB (n=47)	PDD (n=146)	p-value
Age (SD) (years)	73.6±7.9	73.4±8.1	73.7±7.8	0.84
Sex (female), n (%)	82 (42.5)	20 (42.6)	62 (42.3)	0.99
Race	-	-	-	0.47
White, n (%)	157 (81.3)	40 (85.1)	117 (80.1)	
Black or African American, n (%)	32 (16.6)	7 (14.9)	25 (15.1)	
Asian, n (%)	4 (2.1)	0 (0)	4 (4.8)	
Not Hispanic or Latino, n (%)	192 (99.3)	47 (100)	145 (99.4)	0.57
Median years of education (IQR)	14 (12-16)	14 (12-18)	14 (12-16)	0.70

Characteristics of the ADL impairment groups

Principal component analysis performed on all eight ADLs resulted in eight principal components. Components with eigenvalues >1.0 were used for further analysis as they accounted for >50% variance. Component scores for the two principal components were entered into the cluster analysis to obtain clusters. The interpretability of clusters was examined, and it was observed that patients with impairment in six ADLs and patients with impairment in seven ADLs tended to appear together, so they were combined into one group. Performance of hierarchical analysis resulted in the same clustering into three ADL groups: those with impairment in eight ADLs, those with six to seven ADLs impaired, and those with <6 ADLs impaired which were defined as severe, moderate, and mild ADL impairment, respectively. Patients in the mild ADL impairment group had a median duration of dementia of 0.42 years (interquartile range {IQR}: 0.16-0.84); the moderate ADL group had a median duration of dementia of 0.29 years (IQR: 0.10-0.74); and the severe ADL group had a median duration of dementia of 0.26 years (IQR: 0.09-0.65). There was no significant difference in the duration of dementia between the three ADL groups (p = 0.29). 

**Figure 1:**
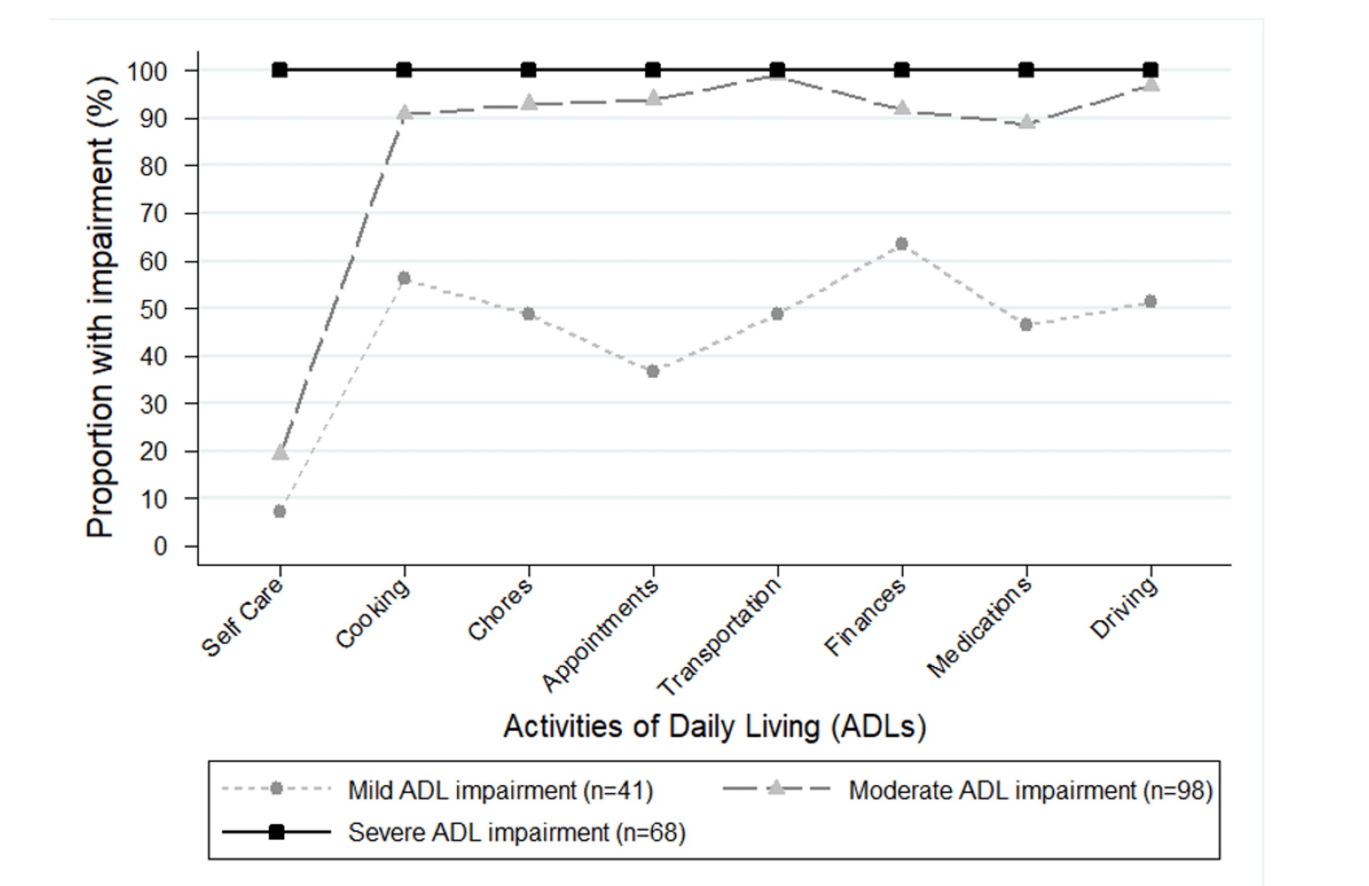
Proportion of participants in each ADL impairment group with impairment on each of eight ADLs. ADL: activities of daily living.

Severe ADL impairment was identified in 32.9% of the sample (n=68), while 47.3% (n=98) were categorized as having moderate ADL impairment, and 19.8% (n=41) had mild ADL impairment. All participants in the severe ADL impairment group were impaired in all ADLs, including self-care, consistent with impairment in basic ADLs. Within the moderately impaired ADL group, 19.4% (n=19) of participants had impairment in self-care, while ≥ 88% had impairment in each of the other seven ADLs. In the mild ADL impairment group, only 7.3% had impairment in self-care, and 36.6-63.4% of participants had impairment in other ADL activities (Figure 1).

Differences in cognitive test performance between the three ADL impairment groups

A comparison of cognitive test scores between the three ADL impairment groups (mild, moderate, and severe) revealed significant differences in performance on Dementia Rating Scale-2 (DRS-2) Total, Trail Making Test A, and Trail Making Test B, and psychomotor processing speed composite score (p<0.001, Table 2). None of the remaining neuropsychological tests showed differences in performance between the ADL impairment groups (Table 2). Of note, among the three ADL impairment groups, there were significant differences in the rate of missing participants in DRS-2 Total, and psychomotor processing speed z-score (Chi-square p<0.05) but not for Trail Making Test A and B. For DRS-2 Total, the greatest rate of missingness was in the mild (46.3%) compared to the moderate (39.8%) and severe (17.6%) ADL impairment groups, while the greatest rate of missingness for psychomotor processing speed z-score was in the severe ADL impairment group (35.3%) compared to the moderate (24.5%) and mild (12.2%) ADL impairment groups.

**Table 2:** Cognitive performance for DRS-2, psychomotor processing speed, and Trail Making Test A and B in each ADL impairment group. Results are reported as means with standard deviations unless otherwise indicated. *These comparisons were statistically significant after correction for false discovery rate at P<0.0067. **The effect size calculated for ANOVA is η2. ^a^Significant difference between ADL mild impairment and ADL severe impairment. ^b^Significant difference between ADL moderate impairment and ADL severe impairment. ADL: activities of daily living, DRS-2: Dementia Rating Scale-2, IQR: interquartile range, RBANS: Repeatable Battery for the Assessment of Neuropsychological Status Form B, SS: scaled score, WAIS: Wechsler Adult Intelligence Scale.

Cognitive domain	Neuropsychological test	ADL impairment						p value	ε^2^
		Mild (n= 41)		Moderate (n= 98)		Severe (n= 68)			
			N (%)		N (%)		N (%)		
Global cognition	DRS-2 Total SS, median (IQR)	5.5 (3-8)	22 (53.6)	6 (3-8)	59 (60.2)	3.5 (2-5)	56 (82.4)	0.002*^a,b^	0.09
Psychomotor processing speed	WAIS-IV Symbol Search SS, median (IQR)	5 (4-7)	19 (46.3)	5 (3-6)	36 (36.7)	4.5 (1.5-5.5)	8 (11.8)	0.23	
	WAIS-IV Coding SS, Mean (SD)	5.3 (2.5)	30 (73.2)	5.68 (2.4)	47 (48.0)	4.14 (1.4)	14 (20.6)	0.10	
	RBANS – Coding z-score (SD)	-3.18 (0.81)	7 (17.1)	-2.35 (0.99)	24 (24.5)	-3.31 (1.3)	29 (42.6)	0.009	
	Composite z-score (SD)	-1.9 (1.1)	36 (87.8)	-1.8 (1.1)	74 (75.5)	-2.9 (1.2)	44 (64.7)	<0.001*^a,b^	0.16**
Executive functions	Trail Making Test A T-score, median (IQR)	31 (19-40)	39 (95.2)	29 (21-36.5)	92 (93.9)	22 (18-29)	60 (88.2)	0.001*^a,b^	0.07
	Trail Making Test B T-score (SD)	27 (16-34)	38 (92.7)	22 (18-39)	91 (92.9)	19 (16-23)	59 (86.8)	0.006*^a,b^	0.06

Post-hoc comparisons revealed that the severe ADL impairment group performed significantly worse on the DRS-2 Total, Trail Making Test A, Trail Making Test B, and psychomotor processing speed z-score compared to the mild and moderate ADL impairment groups (all p<0.05, Table 2). In logistic regression models adjusted for age, sex, education, and depression, DRS-2 Total, Trail Making Test A, Trail Making Test B, and psychomotor processing speed z-score significantly predicted severe ADL impairment (all p<0.05). There were no significant differences in test performances between the mild and moderate ADL impairment groups for these 4 cognitive test scores.

Comparison of Trail Making Test A, Trail Making Test B, and psychomotor processing speed performance based on individual ADL impairment

To further explore the relationship between performance on Trail Making Test A, Trail Making Test B, and psychomotor processing speed and impairment in ADLs, we compared the performance on these assessments in those with and without impairment for each ADL (Tables 3, 4). Participants with impairment in self-care and driving scored significantly worse on Trail Making Test A, Trail Making Test B, and tasks of psychomotor processing speed compared to those participants without impairment (p<0.05). For ADLs other than self-care and driving, Trail Making Test A performance was significantly worse in those with impairment in cooking and medication management; Trail Making Test B performance was significantly worse in those with impairment in cooking and transportation; and psychomotor processing speed was significantly worse in those with impairment in chores, keeping track of appointments, transportation, finances (all p<0.05).

**Table 3:** Relationship between impairment in each ADL and Trail Making Test A and B performance. Results are reported as mean T-scores with standard deviations. ADL: activities of daily living.

ADL	Trail Making Test A T-score (n=191)						Trail Making Test B T-score (n=188)					
	ADL intact		ADL impaired		p value	d	ADL intact		ADL impaired		p value	d
	T-score (SD)	N(%)	T-score (SD)	N(%)			T-score (SD)	N(%)	T-score (SD)	N(%)		
Self-care	30.4 (10.4)	115 (60.2)	25.2 (8.9)	76 (39.8)	<0.001	0.53	25.1 (10.0)	113 (57.1)	21.2 (7.7)	75 (39.9)	0.005	0.42
Cooking	30.7 (11.2)	25 (13.1)	28 (9.9)	166 (86.9)	0.21	0.27	28.5 (12.2)	24 (12.8)	22.8 (8.7)	164 (87.2)	0.005	0.62
Household chores	30.5 (12.7)	24 (12.6)	28.1 (9.7)	167 (87.4)	0.28	0.24	24.4 (12.0)	24 (12.8)	23.4 (8.9)	164 (87.2)	0.60	0.12
Track of appointments	29.3 (11.1)	31 (16.2)	28.2 (9.6)	160 (83.8)	0.58	0.11	26.2 (12.0)	30 (16.0)	23.0 (8.7)	158 (84.0)	0.091	0.34
Transportation	32.1 (13.6)	21 (11.0)	27.9 (9.6)	170 (89.0)	0.069	0.42	28.5 (12.8)	21 (11.2)	22.9 (8.7)	167 (88.8)	0.010	0.60
Finances	29.5 (9.6)	21 (11.0)	28.2 (10.2)	170 (89.0)	0.59	0.12	26.0 (10.7)	21 (11.2)	23.2 (9.2)	167 (88.8)	0.21	0.29
Medication management	33.2 (11.2)	33 (17.3)	27.3 (9.6)	158 (82.7)	0.002	0.59	28.6 (10.7)	33 (17.6)	22.4 (8.7)	155 (82.4)	<0.001	0.69
Driving	32.6 (14.1)	21 (11.0)	27.8 (9.4)	170 (89.0)	0.041	0.48	28.5 (13.0)	21 (11.2)	22.9 (8.6)	167 (88.8)	0.009	0.61

**Table 4:** Relationship between impairment in each ADL and composite processing speed score. Results are reported as mean z-scores with standard deviations. ADL: activities of daily living.

ADL	Processing speed composite z-score (n=154)						
	ADL intact		ADL impaired			p-value	d
	Z-score (SD)	N(%)	Z-score (SD)	N(%)			
Self-care	-2.2 (1.4)	98 (63.6)	-3.3 (1.3)	56 (36.4)	<0.001		0.78
Cooking	-2.1 (1.5)	22 (14.2)	-2.8 (1.4)	132 (85.7)	0.056		0.33
Household chores	-1.8 (1.1)	22 (14.2)	-2.8 (1.5)	132 (85.7)	0.003		0.34
Track of appointments	-2.0 (1.3)	23 (14.9)	-2.8 (1.4)	131 (85.1)	0.014		0.31
Transportation	-1.7 (1.1)	20 (13.0)	-2.8 (1.4)	134 (87.0)	0.001		0.57
Finances	-1.9 (1.5)	19 (12.3)	-2.8 (1.4)	135 (87.7)	0.012		0.54
Medication management	-2.3 (1.5)	29 (18.8)	-2.7 (1.4)	125 (81.2)	0.11		0.39
Driving	-1.8 (1.2)	21 (13.6)	-2.8 (1.4)	133 (86.4)	0.002		0.51

## Discussion

Among tests assessing specific cognitive domains, LBD patients with severe ADL impairment performed worse on a composite psychomotor processing speed z-score, Trail Making Test A, and Trail Making Test B. Our findings indicate that psychomotor processing speed, speeded visuospatial processing, and speeded executive function are associated with ADL impairment in LBD. Previous studies showed that ADL impairment was associated with impairment in attention in Parkinson’s disease with mild cognitive impairment (PD-MCI) and selective/sustained attention and response inhibition in PDD [15,16,25]. With increasing complexity, attention tasks with response inhibition properties are often conceptualized under the executive function domain. In these studies, the more complex sustained attention tasks appear similar to the impaired multitasking task we interpret as a measure of speeded executive function. Consistent with this, other studies have shown that ADL impairment is correlated with impairments in executive function in PD [26], PD-MCI [25], and PDD [27]. Impaired visual-construction function was also associated with ADL impairment in a PDD sample [16]. In summary, our primary findings conform to broader findings showing a relationship between impairment in attention, executive function, and visual construction and ADL impairment in PD.

As expected, patients with severe ADL impairment performed worse on the DRS-2, consistent with previous research indicating that worse performance on the DRS-2 correlated with greater impairment in ADLs among patients with Parkinson’s disease [28]. There was no significant difference in the median DRS-2 score between LBD patients with mild and moderate ADL impairment. This underscores that there can be differences in ADL impairment despite similar global cognitive test performance. Further investigation into the differences between the ADL groups revealed that the major differentiating feature between severe ADL impairment and the other groups was whether patients reported impairment in self-care, which was a measure of all basic ADLs (Figure 1). The difference between the mild and moderate ADL impairment groups was based on the proportion of individuals with impairments in ADLs other than self-care. While this suggests that impairment in self-care was the primary contributor to differences in cognitive performance for the composite psychomotor processing speed z-score, Trail Making Test A and Trail Making Test B, it is important to note that we found that performances on these same cognitive tests were significantly different in those impaired and unimpaired on individual ADLs other than self-care. 

In secondary analyses evaluating whether impairments in individual ADLs were associated with differences in cognitive performance, LBD patients with impaired ability to perform self-care activities and drive had significantly worse performance on the composite psychomotor processing speed z-score, Trail Making Test A and Trail Making Test B. Our findings are consistent with a prior study that found that in patients with mild cognitive impairment (MCI), cognitive processing speed was associated with IADL function and on-road driving performance [29]. In PD patients, both Trail Making Test A and Trail Making Test B test scores have been shown to be correlated with driving performance and errors [30-33] and overall performance on instrumental ADLs [34]. Studies investigating the cognitive domains involved in driving performance in adults with and without cognitive impairment have shown that processing speed, visuospatial function, executive function, and memory correlate to driving ability [35,36]. These findings indicate that psychomotor processing speed, speeded visuospatial functioning, and speeded executive functioning play an important role in ADL impairment and specifically driving ability in LBD, consistent with our findings. Certainly, for many individuals with LBD, motor impairment contributes to ADL performance. However, studies have found that in PDD cognitive impairment independently contributed to the ability to perform instrumental ADLs [37], and in DLB there was also an independent contribution of cognitive impairment to the ability to perform instrumental ADLs [38].

Strengths and limitations

A strength of our study is the relatively large cohort of LBD patients. Even so, we may have been underpowered to detect a significant difference in performance between ADL groups for the all neuropsychological tests included in our analyses. There are limitations to this study based on the fact it was performed using a retrospective, clinical cohort. First, not all participants were administered the same cognitive assessments, and this resulted in different rates of missingness for DRS-2 Total and the processing speed measures. The rates of missingness suggest patients with worse cognition were more likely to be administered the DRS-2 and less likely to be administered processing speed measures. To address the very high rates of missingness for individual processing speed measures, we formed a composite z-score for analyses. Second, comorbidity data were not standardized in patient reports so we were unable to accurately evaluate if differences in comorbidities influenced our findings. Third, our cohort was collected at a tertiary care institution, so our findings may not be generalizable to all LBD patients. Future studies employing prospective recruitment across different sites with a standard protocol could be used to address these limitations. Another limitation is that we examined clinically obtained data and did not use a standardized ADL scale. It may be better to use a standardized ADL assessment such as the Alzheimer’s Disease Cooperative Study-Activities of Daily Living Scale (ADCS-ADL) in future studies [39]. Lastly, cognitive fluctuations, a feature of LBD, may have affected the results, as this remains a difficult aspect of LBD to control for in clinical studies. It was recently reported that in DLB cognitive fluctuations were independently associated with worse daily functioning [40].

## Conclusions

This study expands the current understanding about which cognitive domains contribute to ADL impairment in patients with LBD. We found that worse psychomotor processing speed, speeded visuospatial processing, and speeded executive function were significantly associated with more severe ADL impairment in LBD. Specifically, impairment in self-care activities and driving function were associated with poorer performance in neuropsychological tests for these three domains. These results suggest that interventions aiming to improve processing speed specifically have the potential to improve ADL performance and thus reduce the burden of dementia in LBD for patients and caregivers. To accomplish this, there is a need for further research to understand the pathophysiological underpinnings of impaired processing speed in LBD to inform future therapeutic development.
